# Physiological Arousal and Its Effects on Time Perception: A Systematic Review

**DOI:** 10.1002/wcs.70041

**Published:** 2026-10-10

**Authors:** Luigi Micillo, Nicola Cellini, Giovanna Mioni

**Affiliations:** ^1^ Department of General Psychology University of Padova Padova Italy

## Abstract

Time perception is a fundamental aspect of human cognition, influencing motor coordination, decision‐making, and planning. Unlike sensory modalities such as vision or hearing, which rely on dedicated sensory organs, the perception of time lacks a specific neural substrate, making it highly dependent on internal physiological and psychological states. Research suggests that physiological arousal plays a crucial role in temporal distortions, with pacemaker‐accumulator models proposing that heightened arousal accelerates the internal clock, leading to an overestimation of time. We provide a systematic review of the available literature on the complex relationship between physiological indices of arousal and time distortion. Overall while a relatively consistent association emerged between baseline cardiac autonomic activity and temporal accuracy. When physiological arousal was experimentally manipulated, its relationship with temporal distortion was substantially less consistent. These differences were observed across pain, stress, emotional, and physical‐activity paradigms and appeared to vary according to the characteristics of the eliciting stimulus. This pattern highlights the need to distinguish physiological arousal from the other cognitive processes involved and provides a basis for future research investigating also the other potential explanatory factors in the relationship between arousal and time perception.

## Graphical abstract

This systematic review shows the relationship between physiological arousal and temporal distortions. However, effects vary in magnitude and direction across tasks, cognitive load, stimulus characteristics, and perceived valence. Future studies should integrate physiological measures with subjective arousal to clarify mechanisms.

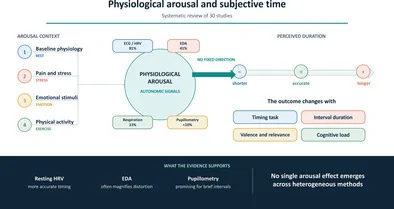

## 1 Introduction

The perception of time is essential for human life, underlying cognitive functions, motor skills, and planning abilities (Buonomano 2017; Grondin 2010; Merchant and de Lafuente 2014; Wearden 2016). From ancient times, philosophers and scientists have studied time's elusive nature and how humans experience its passage (Wittmann 2009). Yet, unlike senses such as sight or hearing, which rely on specific sensory organs, we lack a dedicated organ for perceiving time (Wittmann 2009). This makes time perception dependent on many other ongoing phenomena, suggesting that our sense of time is intrinsically linked to our internal state.

In this way, perceived time can be understood as a reflection of the self, a function shaped by psychological and physiological factors, which can generate a perceptual timing that is not isomorphic with the physical, objective one (Wittmann 2009). A famous example of the non‐neutrality of perceived time is the common saying: “Time flies when we're having fun, and it drags when we're bored.” This simple idiom suggests that emotions can influence our perception of time. Indeed, research shows that emotions can make time feel as if it is more shrunk or expanded (Droit‐Volet and Meck 2007). Similarly, various other phenomena can also induce temporal distortion, such as aging (Visalli et al. 2025), substance use (Arushanyan et al. 2003), clinical conditions (Teixeira et al. 2013; Vatakis and Allman 2015), body temperature (Wearden and Penton‐Voak 1995), and drug addiction (Mioni et al. 2022).

### 1.1 Internal Clock Models

Traditionally, the ability to process temporal information is explained by the presence of a central mechanism, as suggested by the Pacemaker‐Accumulator (PA) models (Gibbon et al. 1984; Treisman 1963, 2013). According to these models, time processing follows three stages: the clock stage, the memory stage, and the decision‐making stage. The first stage, the clock stage, comprises a pacemaker that emits pulses at a regular rate. Pulses pass through the switch and are then stored in an accumulator for the whole duration of the to‐be‐timed event. In the memory stage, the accumulated pulses are stored in working memory and compared with durations stored in long‐term reference memory, which contains past experiences of time intervals. Finally, in the decision‐making stage, information is retrieved from reference memory and compared with the information stored, prompting a behavioral response.

One of the strengths of PA models is that they can explain different temporal distortions depending on the stage of the model that is affected (Lake et al. 2016). For instance, it has been found that temporal overestimation was associated with drug addiction (Mioni et al. 2022; Williamson et al. 2008), body temperature (Wearden and Penton‐Voak 1995), emotional states (Droit‐Volet and Meck 2007), and visual or acoustic flickering (Droit‐Volet and Wearden 2002; Repp et al. 2013). These effects have been explained as arousal‐related temporal distortions (Lake et al. 2016). Specifically, PA models suggest that an increase in arousal alters the rate of the pacemaker, speeding it up. This acceleration leads to the accumulation of a greater number of pulses for a given time, resulting in an overestimation of time (Droit‐Volet et al. 2013; Lake et al. 2016). However, a problem with this model is the definition of arousal. Indeed, in the timing literature, arousal has been conceptualized as “any manipulation that changes the rate of the pacemaker, with an increase in pacemaker rate or internal clock speed equivalent to an increase in arousal” (Lake et al. 2016, 405). As a consequence of this very general and non‐specific definition of arousal in the literature, there is little consistency in how it is manipulated (Cahoon 1969) or measured (Droit‐Volet 2019).

### 1.2 Experimental Methods for Studying Time

Several methods have been developed to assess time perception, each suited to different experimental conditions. Verbal estimation requires participants to express a time interval in clock units, while reproduction involves recreating a presented duration. In temporal production, participants generate an interval based on a given time specification, often using taps or button presses (Mioni 2018). In the comparison method, participants are asked to judge the relative length of temporal intervals, either with a fixed standard (reminder method) or varying trials (roving method) (Bausenhart et al. 2018). A variation, the single‐stimulus method, requires participants to categorize an interval as “short” or “long,” as seen in bisection and temporal generalization tasks (Penney and Cheng 2018). In the finger tapping task, participants are asked to tap at a speed that feels natural and comfortable for them, ensuring that the interval between each tap is as consistent as possible (Hammerschmidt et al. 2021) or, in a different version of the task, to tap at a predetermined rhythm (Perbal et al. 2003).

### 1.3 Physiological Arousal and Its Measurements

A recent review of the literature reveals a vast body of research on arousal, with approximately 50,000 studies addressing this concept in various contexts (Sabat et al. 2025). Despite this extensive research, a clear and precise definition of arousal is still lacking (Sabat et al. 2025). The Encyclopedia of Behavioral Medicine (Gellman 2020) defines arousal as the state of being activated, either psychologically or physiologically, involving changes such as increased heart rate (HR) and blood pressure (Niven and Miles 2013). This definition suggests that arousal is closely linked to the autonomic nervous system (ANS), which has a key role in regulating the physiological dimension of arousal. The ANS consists of two branches: the sympathetic nervous system (SNS), which controls the body's “fight or flight” responses, and the parasympathetic nervous system (PNS), which helps restore equilibrium. During heightened arousal, the SNS becomes dominant, increasing HR, respiration, blood pressure, pupil dilation, and skin conductance, reflecting increased physiological activation. The PNS, conversely, promotes relaxation and recovery (Berntson et al. 2007).

Several methods exist to measure arousal and its physiological correlates, providing valuable insights into its underlying mechanisms. For example, heart rate variability (HRV) measures fluctuations in the intervals between heartbeats, offering an indicator of ANS balance and stress responsiveness (Quigley et al. 2024). Skin conductance, or electrodermal activity (EDA), reflects SNS activation through changes in sweat gland activity (Critchley 2002). Pupillometry, which tracks pupil size, is another non‐invasive method that serves as an indicator of arousal, as pupil constriction and dilation correlate with PNS and SNS activity, respectively (Skaramagkas et al. 2023). The use and combination of these techniques offer a more comprehensive understanding of arousal and its effects on cognition and behavior (Ishikawa 2023).

To sum up, while the definition of arousal remains elusive despite extensive research, it plays a significant role in both psychological and physiological processes, particularly through its influence on the ANS. This systematic review critically analyses the current literature on the role of physiological arousal in temporal distortion, focusing on the mechanisms through which arousal influences time perception.

### 1.4 Aims and Research Inquiries

Despite extensive research on both time perception and arousal, the relationship between these constructs remains unclear, particularly with the various physiological indices used to measure arousal (e.g., HRV, skin conductance, and pupillometry).

## 2 Methods

### 2.1 Literature Search

A systematic search strategy was performed following the PRISMA recommendations (Page et al. 2021; Rethlefsen et al. 2021). Firstly, we consulted PsycInfo, PubMed and Web of Science using the terms “Time Perception,” “Timing,” “Time Production,” “Time Reproduction,” “Time Estimation,” “Explicit Timing,” “Implicit Timing” in combination with “Psychophysiology,” “Physiological Arousal,” “Heart Rate,” “Heart Rate Variability,” “HR,” “Skin Conductance,” “SCL,” “Electrodermal Activity,” “EDA,” “Galvanic Skin Response,” “GSR,” “Skin Resistance,” “Vagal Activity.”

Reference lists from published reviews, books, and chapters were additionally checked to identify studies that the databases' search might have missed. A literature search was conducted independently by LM. The latest search was carried out in December 2024 without any time restriction. Articles selection was performed by LM. Any doubts or uncertainties were resolved by discussion with GM and NC until a consensus was reached. A total of 3981 potentially relevant studies were found, and 3286 records remained after duplicates were removed. Then, each abstract was evaluated for eligibility with AS Review Lab (ASReview LAB developers 2024; Van De Schoot et al. 2021), identifying 137 articles as eligible. A total of 114 articles did not meet the inclusion criteria; therefore, 23 papers were included in the present review. Additionally, the reference lists of the included articles were reviewed to identify other relevant studies. Out of a list of 15 potentially eligible articles, 7 were added, bringing the total to 30 papers for review (Figure 1).

**FIGURE 1:**
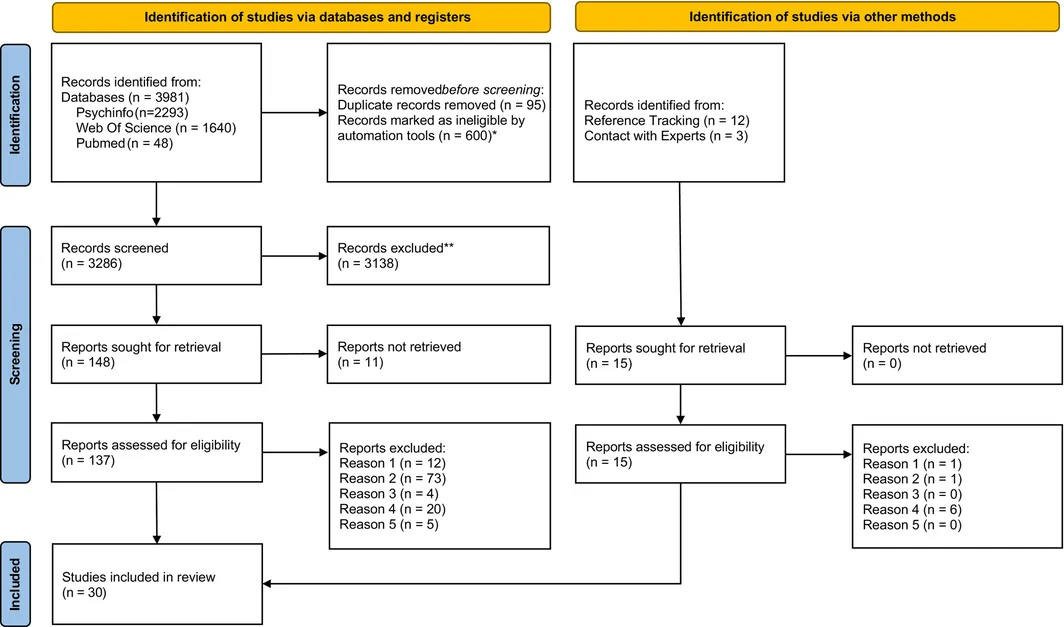
Flow chart of the study screening process. PRISMA 2020 flow diagram including searches of databases, registers, and other sources. *600 records were marked as ineligible by using the automatic categorization in Zotero. ** The screening process was carried out using AS review lab.

### 2.2 Inclusion Criteria

Articles were selected according to the following criteria:

1. The documents must be research articles published in peer‐reviewed journals.
2. Published articles had to report both measurements of temporal processing and recording of physiological arousal (i.e., electrocardiogram, skin conductance, pupillometry).
3. The included articles must be written in English, French, Spanish, or Italian.
4. The studies included in this work should have tested temporal abilities and physiological activity in healthy adults in order to avoid effects of other possible confounders that lie under the definition of arousal in timing literature.
5. Arousal induction should not be delivered via drugs, psychoactive substances, or other factors that may alter arousal while also affecting other physiological or cognitive systems, as this could lead to confounding effects and distortions in the results.

### 2.3 Organization of the Works

Once the selection of studies was completed, we observed significant heterogeneity in terms of the types of arousing manipulations and measurements, temporal tasks, and duration used. To simplify the presentation for the reader, we organized the studies into four main categories based on the specific manipulation employed (or not) to alter arousal levels.

The first category includes studies that did not directly manipulate the arousal state, such as those that used physiological activity at rest or spontaneous physiological fluctuations during temporal tasks as measures of arousal.

The second category encompasses studies that manipulated arousal through stressful or painful experiences. Within this category, we further distinguish between studies that used pain to induce arousal alterations and those that induced stress in participants.

Given the significant overlap between “Emotion” and “Arousal” in the timing literature, we created a third category for studies that both manipulated arousal through emotional induction and recorded it.

The final category includes studies that used physical activity to alter participants' arousal states.

### 2.4 Risk of Bias

The methodological quality of the included studies was assessed using the JBI Critical Appraisal Tools (Moola et al. 2015), which evaluate how effectively each study addresses potential sources of bias in its design, conduct, and analysis. Each criterion was rated as “yes” (meeting the quality standard), “no” (indicating potential bias), “unclear,” or “not applicable.”

For each study, an overall score was calculated as the proportion of “yes” responses relative to the number of applicable criteria. Studies were categorized by risk of bias as high (< 49%), moderate (50%–69%), or low (> 70%). Figure 2 presents a summary of the risk‐of‐bias assessment for each work included.

**FIGURE 2:**
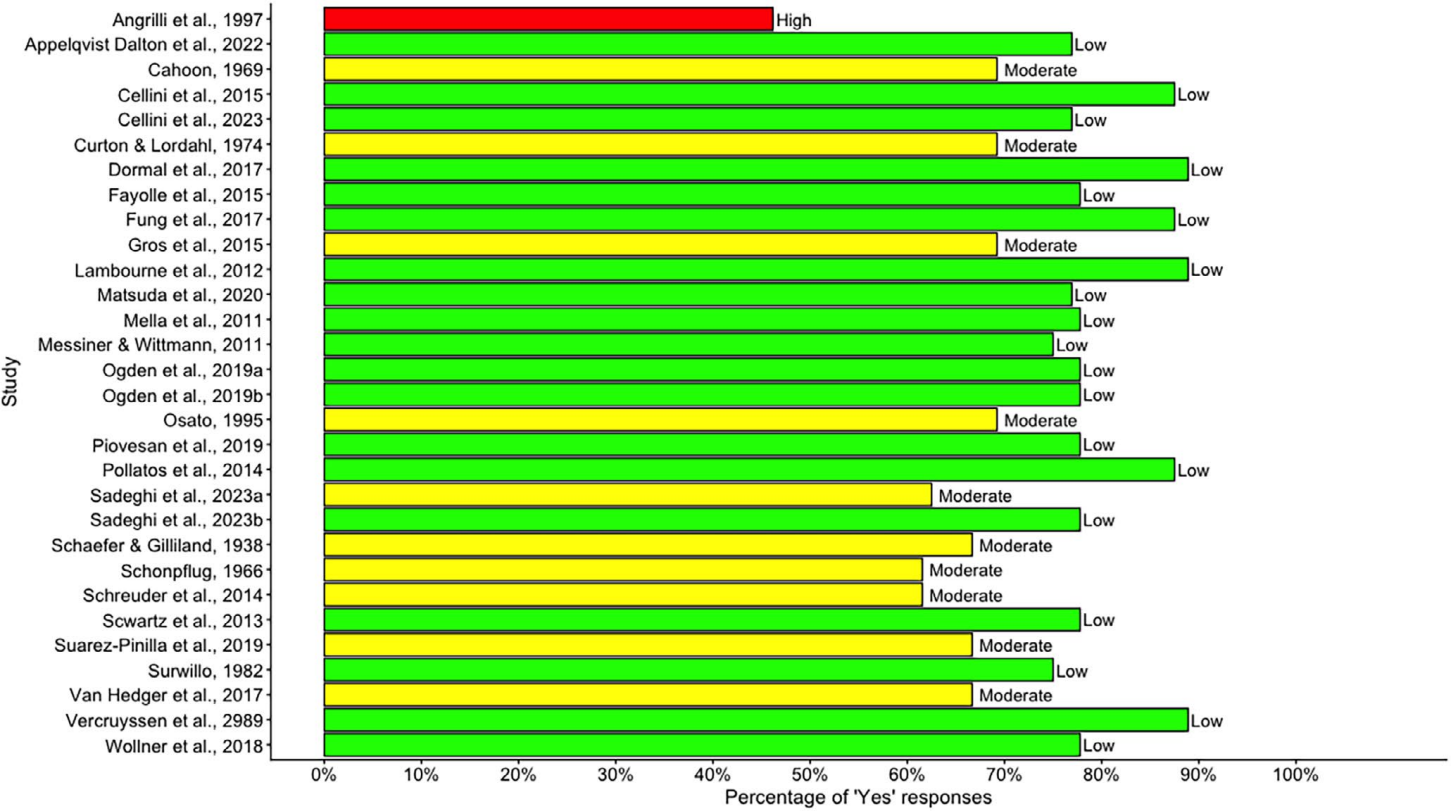
Summary of the risk‐of‐bias assessment across studies.

## 3 Results

### 3.1 Studies With no Direct Manipulation of Arousal

According to Wittmann (2009, 2013), temporal experience is deeply intertwined with bodily experiences. Therefore, physiological rhythms can be a strong base for constructing subjective temporal representations (Craig 2009). Following this hypothesis, we gathered studies that did not actively manipulate participants' arousal (Table 1) but investigated the relationship between resting autonomic activity and temporal performance, or investigated the relationship between physiological activation and temporal performance by recording participants' physiological activation while performing temporal tasks.

**TABLE 1:** Studies included in the no manipulation category.

Article	Sample *N* (Females)	Age M (SD)	Study design	Country	Recording	Task	Durations	Arousal index	Effects arousal	Time index	Effects time
Cellini et al. (2015)	30 (21)	24.18 (2.1)	Within	Italy	ECG	Time bisection	0.3–0.9 s	HRV	↑	CE	+
								TP	↑	CE	+
						Finger tapping	Free	HRV	↑	ITI	+
								TP	↑	ITI	+
							1 s	HRV	↑	AE	+
								TP	↑	AE	+
					ICG	Time bisection	0.3–0.9 s	PEP	↑	CE	+
						Finger tapping	Free			ITI	+
							1 s			AE	−
Fung et al. (2017)	120 (63)	25 (ND)	Within	Australia	ECG	Time reproduction	2–15 s	HR	↑	CE	−
										CV	−
										*α*	+
										*β*	−
								LF‐HRV	↑	CE	−
										CV	−
										*α*	+
										*β*	−
								HF‐HRV	↑	CE	+
										CV	−
										*α*	+
										*β*	−
								RMSSD	↑	CE	+
										CV	−
										*α*	+
										*β*	−
Meissner and Wittmann (2011)	31 (20)	ND	Within	ND	ECG	Time reproduction	8–20 s	RR Interval	↑	PCE	+
					EDA		8–20 s	SCL	↓	PCE	/
Pollatos et al. (2014)	23 (18)	23.8 (3.1)	Within	Germany	ECG	Time reproduction	0.5–40 s	RMSSD	↑	AE	+
Sadeghi, Wittmann, et al. (2023)	45 (29)	ND	Within	USA	ECG	Time bisection	0.08–0.18 s	PSHR	↓	TB	>
Surwillo (1982)	92 (0)	ND	Within	ND	ECG	Time production	30–180 s	HR	↑	RE	<

Abbreviations: AE, absolute error |(subjective‐objective)|; CE, constant error (subjective‐objective); CV, coefficient of variation; ECG, electrocardiogram; EDA, electrodermal activity; HF‐HRV, high‐frequency heart rate variability; HR, heart rate; HRV, heart rate variability; ICG, impedance cardiography; ITI, inter‐tap interval; LF‐HRV, low‐frequency heart rate variability; M, mean; N, numerosity of the sample; ND, non‐disclosed; PCE, proportional constant error ((subjective‐objective)/objective); PEP, pre‐ejection period; PSHR, pre‐stimulus heart rate; RE, raw estimate; RMSSD, root mean square of the successive differences; RR, interval between two R peaks; SCL, skin conductance level; SD, standard deviation; TB, temporal bias; TP, total power; α, scale parameter (values ≠ 1 indicate time perception bias); β, exponent β < 1 indicates concavity, β > 1 indicates convexity, and β = 1 indicates linearity; ↑, Increase; ↓, Decrease; +, More Accurate; −, Less Accurate; /, No Effect; <, Underestimation; >, Overestimation.

Considering the studies that investigated the relationship between resting autonomic activity and temporal performance, baseline cardiac activity has been repeatedly linked to temporal accuracy. In Surwillo (1982), participants were asked to produce time intervals (30, 60, and 180 s—Figure 3A) before undergoing an electrocardiogram (ECG) resting state recording. Correlational analyses showed that higher resting HR was associated with lower temporal accuracy for short durations. Years later, Pollatos et al. (2014) asked their participants to complete a time reproduction task (0.5–40 s—Figure 3A), and their performance was positively correlated with PNS activity at rest, as indexed by Root Mean Square of Successive Differences (RMSSD), an HRV index derived from ECG recording. Similarly, Cellini et al. (2015) correlated participants' performances in time bisection (0.3–0.9 s, Figure 3A) and finger tapping tasks (1 s vs. free tempo) with HRV indices computed from a 5‐min resting ECG. The authors observed that higher PNS activity at rest, as indexed by the high‐frequency (HF‐HRV) and the total power of HRV, was associated with a more accurate performance in both the time bisection and 1‐s finger tapping task. Consistently, lower sympathetic activity, as indexed by longer PEP, was associated with greater absolute error. In Fung et al. (2017), participants were asked to complete a time reproduction task (2–15 s—Figure 3A) after recording their resting cardiac activity. They assessed SNS and PNS activity by computing with Low‐Frequency HRV (LF‐HRV) and HF‐HRV. The authors reported that both LF‐HRV and HF‐HRV (*p* = 0.056) were negatively associated with lower temporal accuracy. However, these effects should be discussed by reinterpreting the role of resting states LF‐HRV as an index of baroreflex‐mediated cardiac PNS activity rather than cardiac SNS activity (Shaffer and Ginsberg 2017; Quigley et al. 2024).

**FIGURE 3:**
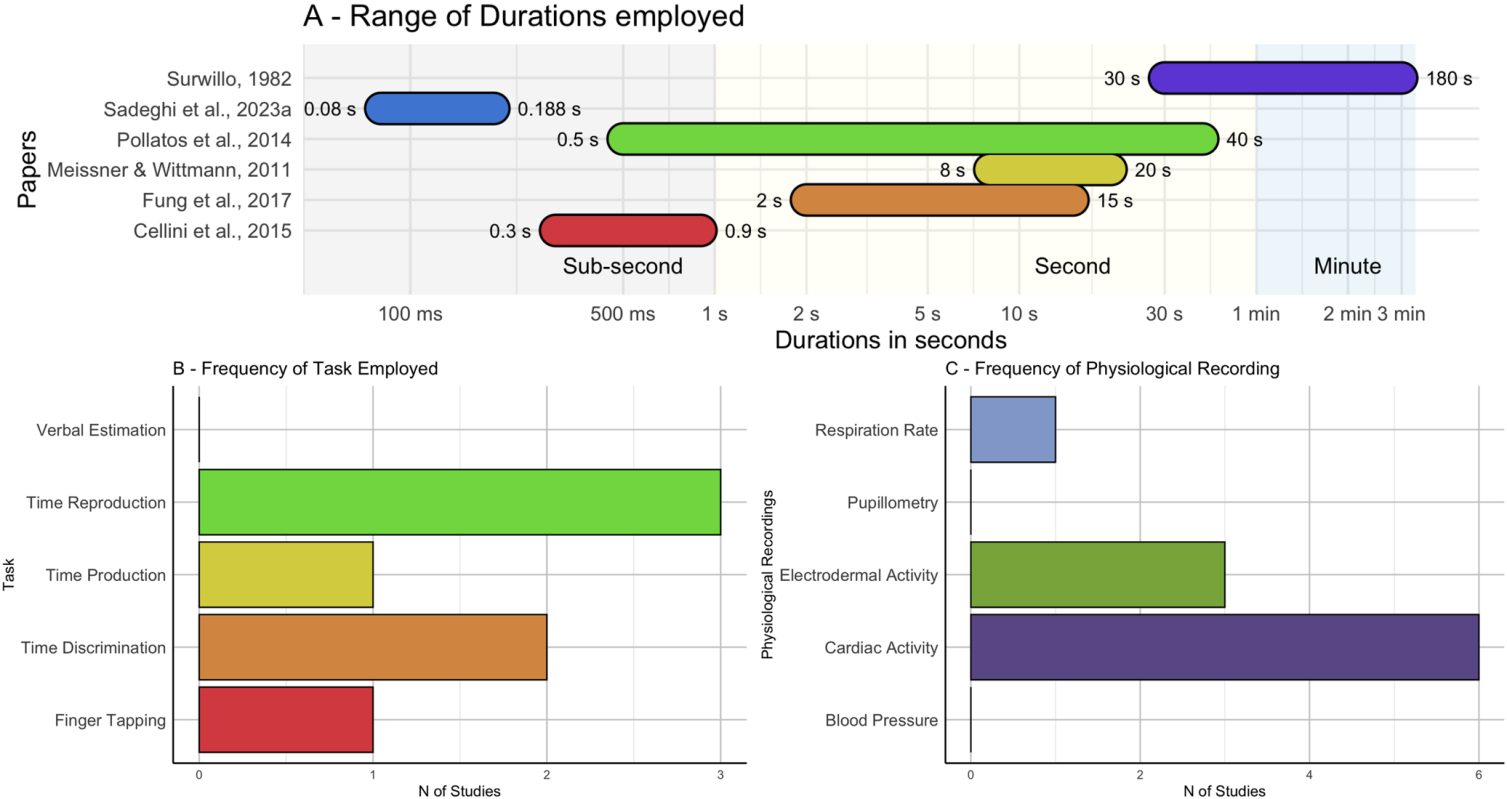
Duration ranges, frequency of tasks, and physiological measures in the analyzed studies. (A) Durations range from subsecond to minutes. (B) Number of studies using different timing tasks. (C) Frequency of physiological measures assessed.

Concerning studies investigating the relationship between physiological activation and temporal performance by recording participants' physiological activation *during task execution*, we cannot draw any conclusion. For instance, Meissner and Wittmann (2011) asked participants to reproduce 8, 14, or 20 s (Figure 3A) while recording ECG, EDA, and Respiration Rate. They observed an increase in second‐to‐second change in cardiac periods (i.e., the temporal distance between two consecutive heartbeats) that was positively associated with temporal accuracy. Sadeghi, Wittmann, et al. (2023), using a time bisection task (0.8–1.88 s—Figure 3A) synchronized with participants' heartbeats, showed that the perception of time intervals varied depending on the preceding heartbeat: a shorter heartbeat before the sound led participants to perceive the interval as longer, while a longer preceding heartbeat made it seem shorter. Pre‐stimulus HR also influenced temporal perception, with a lower HR before stimulus onset linked to a bias toward perceiving durations as longer. The methodological differences between these two studies do not allow us to conclude what concerns the relationship between arousal and time perception when arousal is measured during the execution of the temporal task.

To sum up, the studies presented above highlight a consistent relationship between cardiac PNS activity at rest and temporal accuracy. Interestingly, this relationship between baseline cardiac activity and time perception is observed not only across different durations (Figure 3A) but also across a range of temporal tasks (Figure 3B), e.g., time bisection task, finger tapping task, time reproduction task, and time production task. This suggests a consistent relationship between baseline physiological activation and time perception across different temporal tasks, highlighting the robustness of the effect. However, this relationship loses consistency when physiological arousal is assessed during task execution.

### 3.2 Studies That Manipulated Arousal With Painful or Stressful Experiences

#### 3.2.1 Painful Experiences

One of the possible ways of altering the arousal state is by delivering pain or shocks (Kyle and McNeil 2014). We found two studies in the literature (Fayolle et al. 2015; Piovesan et al. 2019) that analyzed temporal alterations by delivering actual shocks or pain to modulate physiological activation (Table 2A). These studies suggest that the effect of pain‐induced arousal on time perception is somehow modulated by the relevance of the painful stimulus. Specifically, Fayolle et al. (2015) delivered electrical shocks on 67% of trials of a time bisection task (0.2–8 s—Figure 4A) while measuring participants' EDA. They observed no effect of shocks on temporal sensitivity. However, participants consistently perceived trials involving shocks, which were associated with higher EDA, as being longer than those without shocks. Piovesan et al. (2019) asked participants to verbally estimate the duration of an electrical shock (0.242–1.296 s—Figure 4A) while ECG and EDA were recorded. Participants judged the durations of high‐pain shocks as significantly longer than those of low‐pain shocks. Higher skin conductance levels (SCL) were associated with longer perceived durations. However, HF‐HRV showed no significant relationship with time perception. In a second experiment, Piovesan et al. (2019) used thermal stimuli to modulate arousal levels. In this case, the shocks were not as inherently tied to the timing task since participants had to judge the duration of white circles (0.242–1.296 s—Figure 4A) presented during the thermal stimulation. This time, participants did not perceive significant differences in duration estimates, nor did SCL or HF‐HRV show a meaningful relationship with time perception. Piovesan et al. (2019) suggested that the differences observed between their two experiments were due to the relevance of the painful stimulus to the timing task. In the first experiment, the painful stimulus itself was the target of timing, whereas in the second, participants were asked to time a neutral stimulus. However, the findings from Fayolle et al. (2015) challenge this interpretation, as participants in their study also timed a neutral stimulus while experiencing pain, yet still showed altered time perception.

**TABLE 2:** Studies included in the shock and pain category.

Article	Sample *N* (females)	Age M (SD)	Study design	Country	Arousal manipulation	Recording	Task	Durations	Arousal index	Effects arousal	Time index	Effects time
A. Painful experiences												
Fayolle et al. (2015)	60 (48)	19.41 (4.8)	Between (duration) within (manipulation)	France	Electric Shock	EDA	Time Bisection	0.2–8 s	EDA	↑	BP	>
Piovesan et al. (2019)	40 (30)	26.20 (3.91)	Within	United Kingdom	Electric shock	EDA	Verbal Estimation	0.242–1.296 s	SCL	↑	RE	>
	31 (26)	22.23 (4.74)	Within	United Kingdom	Thermal shock	EDA	Verbal Estimation	0.242–1.296 s	SCL	↑	RE	/
Cahoon (1969)	40 (ND)	ND	Between (chronic arousal) within (manipulation)	ND	Threat of shock	ECG	Verbal Estimation	36 s	HR	↑	RE	>
							Time Production	22 s	HR	↑	RE	>
							Finger Tapping	1 s	HR	↑	ITI	>
						PZG	Verbal Estimation	22 s	RSP	↑	RE	/
							Time Production	36 s	RSP	↑	RE	/
							Finger Tapping	1 s	RSP	↑	ITI	/
B. Stressful experiences												
Cellini et al. (2023)	38 (28)	22.89 (2.5)	Between	Italy	PASAT	ECG	Time Bisection	0.3–0.9 s	HR	↑	CE	+
							Finger Tapping	Free	HR	↑	ITI	/
						EDA	Time Bisection	0.3–0.9 s	GM	/	CE	+
							Finger tapping	Free	GM	/	ITI	/
Matsuda et al. (2020)	36 (23)	21.4 (ND)	Within	Japan	Concealing	EDA	Time discrimination	1.8–2.2	SCR	↑	DB	>
Osato et al. (1995)	60 (60)	ND	Between	Japan	Evaluative Instructions	ECG	Time reproduction	4–16 s	HR	↑	RE	<
							Verbal estimation	4–16 s	HR	↑	RE	>
Sadeghi, Daziano, et al. (2023)	41 (19)	22.6 (5.5)	Within	USA	Crowd density (VR)	PPG	Verbal estimation	60–80s	IBI	/	CE	>
Van Hedger et al. (2017)	42 (16)	19.95 (1.3)	Within	USA	TSST + IAPS Pos	ECG	Time reproduction	0.4–4.15 s	HR	↑	Δ post‐pre exposure	>
					TSST + IAPS Neg					↑		>
					TSST + IAPS Neu					↑		>
					TSST + IAPS Pos				HRV	/		/
					TSST + IAPS Neg					/		/
					TSST + IAPS Neu					/		/
					TSST + IAPS Pos	ICG			PEP	↓		/
					TSST + IAPS Neg					↓		>
					TSST + IAPS Neu					↓		/

Abbreviations: BP, bisection point; CE, constant error (subjective—objective); DB, discrimination bias ((number of “long” responses − number of “short” responses)/total number of responses); ECG, electrocardiogram; EDA, electrodermal activity; GM, global mean; HR, heart rate; HRV, heart rate variability; IAPS, international affective picture system; IBI, inter‐beat interval; ICG, impedance cardiography; ITI, inter‐tap interval; M, mean; N, numerosity of the sample; ND, non‐disclosed; Neg, negative valence; Neu, neutral valence; PASAT, paced auditory serial addition test; PEP, pre‐ejection period; PPG, photoplethysmography; Pos, positive valence; PZG, piezoelectrography; RE, raw estimate; RSP, respiration rate; SCL, skin conductance level; SCR, skin conductance response; SD, standard deviation; TSST, Tirer social stress test; VR, virtual reality; Δ, difference in RATIO (objective/subjective); ↑, Increase; ↓, Decrease; +, More Accurate; /, No Effect; <, Underestimation; >, Overestimation.

**FIGURE 4:**
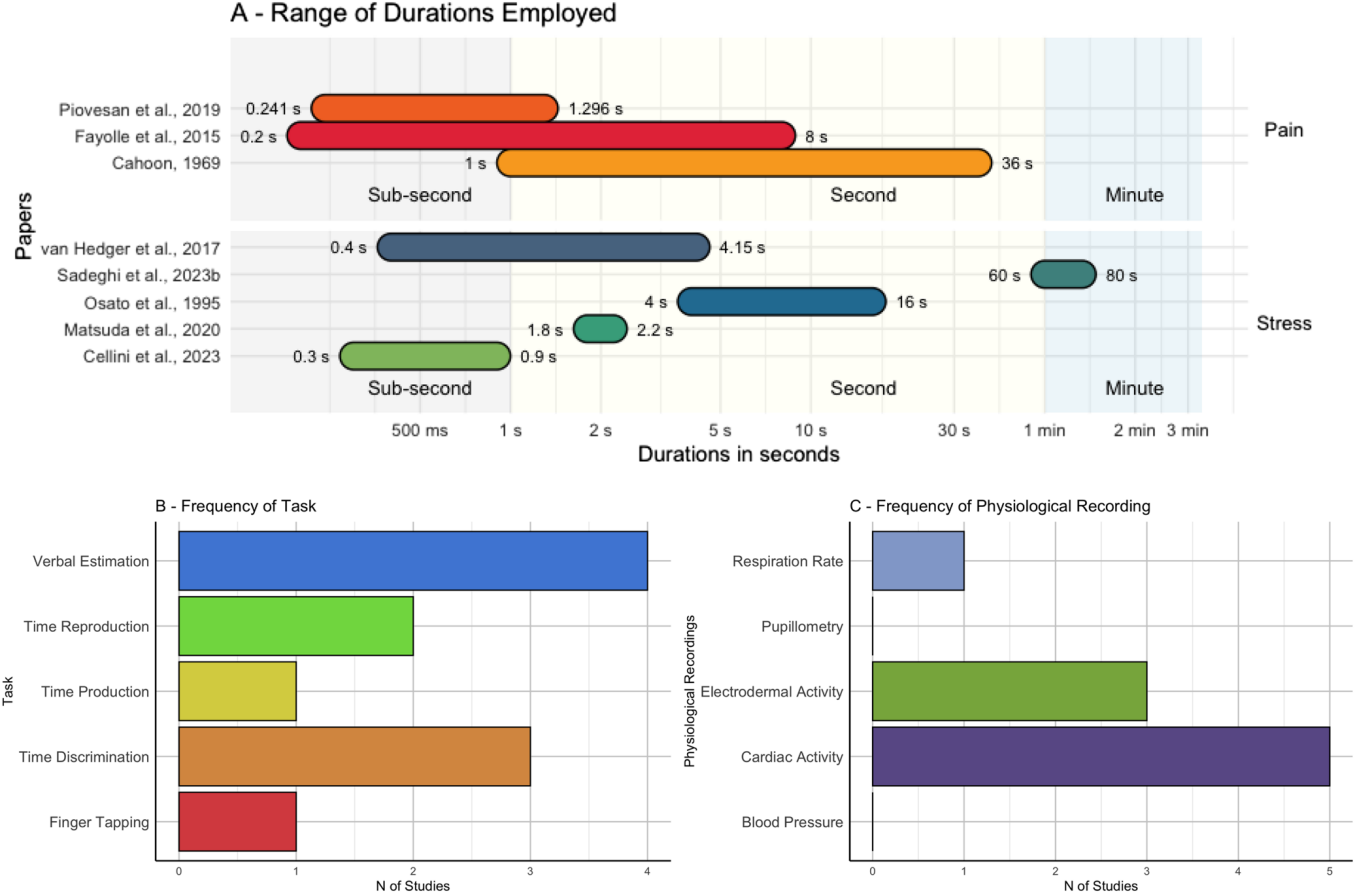
Duration ranges, frequency of tasks, and physiological measures in the analyzed studies. (A) Durations range from subsecond to minutes. (B) Number of studies using different timing tasks. (C) Frequency of physiological measures assessed.

Together, these two studies suggest that the nature of the painful stimulus itself may be an important modulator of the effect of pain‐induced arousal on time perception. Consistent with this idea, Cahoon in 1969 threatened participants, without actually delivering, with receiving an electric shock in case they did not respond in time to a post‐trial warning. The results revealed that the threat of shock increased participants' HR and was positively associated with verbal time estimation, time production, and finger‐tapping performance.

#### 3.2.2 Stressful Experiences

It is a common experience that stressful events, such as public speaking or performing a complex task, can cause noticeable changes in arousal. Indeed, social stressors are particularly effective for examining these effects due to their inherent ties to temporal processing and their significant influence on human psychophysiology (Van Hedger et al. 2017). For example, social stressors, including public speaking (Van Hedger et al. 2017) and evaluative instructions (Osato et al. 1995), induce measurable changes in both HR and temporal judgments (Table 2B).

Van Hedger et al. (2017) discovered that blending emotion with a modified version of the Trier Social Stress Test (TSST—Kirschbaum et al. 1993) heightened SNS activity, indicated by Pre‐Ejection Period (PEP), while diminishing PNS activity (HF‐HRV). Additionally, this combination induced distortions in temporal reproduction for durations of 0.4 to 4.15 s (Figure 4A). Specifically, emotional stimuli were perceived to last longer following stress induction, emphasizing a relationship between stress and negative emotions. Interestingly, only the change in PEP was significantly correlated with time distortion for negative images, and post‐TSST HR was positively correlated with time distortion for negative and positive images. Osato et al. (1995) examined the effects of stressful experiences, induced through evaluative instructions, on HR, memory, verbal estimation, and reproduction tasks (4–16 s—Figure 4A). The results showed that the effects of the manipulation on participants' HR were modulated by at‐rest (baseline) cardiac activity. Indeed, initial high‐HR individuals experienced further HR increases under standard instructions, while initial moderate‐HR individuals exhibited increases under evaluative instructions. In contrast, initial low‐HR participants remained unaffected by the instruction type. In this experiment, reproduction tasks resulted in underestimations of time intervals, with longer estimates for evaluative instructions at 16 s, while verbal estimates generally led to overestimations. Although the authors suggest an overlapping effect of arousal and time perception, no significant correlations were observed between HR at rest and during the tasks with temporal performance. Matsuda et al. (2020) explored how the act of concealing an item influences time perception and the associated physiological arousal. They found that participants who were asked to steal and conceal an item overestimated time in a temporal generalization task (1.8–2.2 s—Figure 4A). Physiologically, it was found that participants in the concealing group exhibited higher SCL compared to a control group during the timing task.

The results of these studies showed that although social stressors have a clear heightening effect on physiological arousal, a similar pattern in timing abilities is not observed. Besides social stress, other authors have explored how cognitive stressors influence temporal abilities. For instance, Cellini et al. (2023) manipulated arousal using a serial addition test. Participants were assigned to one of two tasks: the Paced Auditory Serial Addition Test (PASAT; stress condition—Gronwall 1977) or the Paced Auditory Number Reading Task (PANRAT; control condition—Tanosoto et al. 2015) before a time bisection task (0.3–0.9 s, Figure 4A). As expected, participants in the stress condition reported a higher perceived stress and showed higher HR and EDA levels. Moreover, they displayed greater accuracy in the bisection task after completing the stress‐inducing PASAT than after the less challenging PANRAT. Sadeghi, Daziano, et al. (2023) induced stress by manipulating crowd density in a Virtual Reality subway journey whose durations (60–8 s—Figure 4A) were the to‐be‐timed event. They found that increased crowd density was associated with longer perceived time travel. Although crowded spaces were associated with subjective feelings of discomfort, changes in interbeat intervals were correlated with the perceived travel time, independent of the crowd density. Specifically, a greater cardiac deceleration was associated with longer perceived travel time.

Overall these studies highlight that the effects of stress on temporal processes are not uniform but rather depend on the type of stress (e.g., social vs. cognitive) and other factors such as the cognitive load of the stressful manipulation (Cellini et al. 2023), the subjective perception of the experience (Matsuda et al. 2020), and the relevance that the stressor has for the temporal task (Piovesan et al. 2019). Another possible explanation for the inconsistency of those results may lie in the strong heterogeneity of the tasks employed (Figure 4B) and of the physiological indexes used (Figure 4C). The specificity of the demands of each temporal task and its association with the demands of the manipulation employed may have contributed to inconsistent or non‐generalizable results.

### 3.3 Studies With Arousal Manipulation Made With Emotional Stimuli

A vast literature reports that emotional experiences can alter time perception (see Droit‐Volet and Meck 2007; Lake et al. 2016). Among the studies investigating the effect of emotions on time perception, although many have referred to arousal mechanisms to explain their findings, only a limited number have recorded physiological measures (Droit‐Volet 2019). These studies employed various emotional stimuli, including images (Table 3A), sounds (Table 3B), videos (Table 4), and even multi‐stimuli presentations (Table 5). The following section presents studies based on the types of emotional stimuli utilized.

**TABLE 3:** Studies that used emotional images and sounds included in the emotion manipulation category.

Article	Sample *N* (Females)	Age M (SD)	Study design	Country	Arousal manipulation	Recording	Task	Durations	Arousal index	Effects arousal	Time index	Effects time
A. Emotional images												
Angrilli et al. (1997)	53 (ND)	ND	Between (task) within (manipulation)	Italy	IAPS—Low Negative	ECG	Time Reproduction	2–6 s	HR	↓	PCE	<
					IAPS—High Negative					↓		<
					IAPS—Low Positive					↑		<
					IAPS—High Positive					↑		<
					IAPS—Low Negative	EDA			SCR	↑		<
					IAPS—High Negative					↑		<
					IAPS—Low Positive					↑		<
					IAPS—High Positive					↑		<
					IAPS—Low Negative	ECG	VAS	2–6 s	HR	↓	PCE	<
					IAPS—High Negative					↓		<
					IAPS—Low Positive					↑		<
					IAPS—High Positive					↑		<
					IAPS—Low Negative	EDA			SCR	↑		<
					IAPS—High Negative					↑		<
					IAPS—Low Positive					↑		<
					IAPS—High Positive					↑		<
Ogden, Henderson, McGlone, and Richter (2019)	50 (37)	20.68 (3.37)	Within	United Kingdom	IAPS—Low Negative	ICG	Verbal estimation	0.2–0.8 s	PEP	/	Δ emotional‐neutral	>
					IAPS—High Negative					↑		>
					IAPS—Low Positive					/		/
					IAPS—High Positive					/		/
Ogden, Henderson, Slade, et al. (2019)	30 (18)	25.30 (3.63)	Within	United Kingdom	IAPS + Breathing—Negative	ICG	Verbal estimation	0.2–0.8 s	PEP	/	RATIO	<
					IAPS + Breathing—Negative	ECG			HRV	↑		<
B. Emotional sounds												
Mella et al. (2011)	19 (10)	26 (2.45)	Within	ND	IADS—High Arousal/Attention Time	EDA	Time discrimination	1.8–2.2	SCR	/	DB	/
					IADS—Low Arousal/Attention Time					/		/
					IADS—High Arousal/Attention Emotion					↑		>
					IADS—Low Arousal/Attention Emotion					/		/

Abbreviations: DB, discrimination bias ((number of “long” responses − number of “short” responses)/total number of responses); ECG, electrocardiogram; EDA, electrodermal activity; HR, heart rate; HRV, heart rate variability; IADS, international affective digitized sounds; IAPS, international affective picture system; ICG, impedance cardiography; M, mean; N, numerosity of the sample; ND, non‐disclosed; SD, standard deviation; PEP, pre‐ejection period; RATIO (subjective/objective); PCE, proportional constant error ((subjective‐objective)/objective); SCR, skin conductance response; VAS, visual analogue scale; Δ, difference in total estimate scores; ↑, Increase; ↓, Decrease; /, No Effect; <, Underestimation; >, Overestimation.

**TABLE 4:** Studies that used videos included in the emotion manipulation category.

Article	Sample *N* (Females)	Age *M* (SD)	Study design	Country	Arousal manipulation	Recording	Task	Durations	Arousal index	Effects arousal	Time index	Effects time
Appelqvist‐Dalton et al. (2022)	30 (ND)	20.7 (1.5)	Between	USA	Videos—Audio Video	Oculometry	Verbal estimation	5–13 s	Pupil dilation	/	PCE	+
					Videos—Video Only					/		<
					Videos—Audio Only					/		<
Suárez‐Pinilla et al. (2019)	55 (40)	21.4 (ND)	Within	United Kingdom	Videos—Indoor	ECG	VAS	1–64 s	HR	/	z score	>
					Videos—Outdoor City				HR	/		>
					Videos—Outdoor Green				HR	/		>
					Videos‐ Indoor	Oculometry			Saccade density	/		>
					Videos—Outdoor City				Saccade density	/		>
					Videos—Outdoor Green				Saccade density	/		>
Wöllner et al. (2018)	45 (21)	23.59 (4.31)	Within	Germany	Video + Speed—Audio+Video (Slow)	ECG	Verbal estimation	2.67–40 s	HR	↑	PCE	<
					Video + Speed—Video Only (Slow)					↑		<
					Video + Speed—Audio+Video (Regular)					↑		<
					Video + Speed—Video Only (Regular)					↑		<
					Video + Speed—Audio+Video (Slow)	EDA			SCR	↑		<
					Video + Speed—Video Only (Slow)					↑		<
					Video + Speed—Audio+Video (Regular)					↑		<
					Video + Speed—Video Only (Regular)					↑		<
					Video + Speed—Audio+Video (Slow)	Oculometry			Pupil diameter	↑		<
					Video + Speed—Video Only (Slow)					↑		<
					Video + Speed—Audio+Video (Regular)					↑		<
					Video + Speed—Video Only (Regular)					↑		<
					Video + Speed—Audio+Video (Slow)	PZG			RSP	↑		<
					Video + Speed—Video Only (Slow)					↑		<
					Video + Speed—Audio+Video (Regular)					↑		<
					Video + Speed—Video Only (Regular)					↑		<

Abbreviations: ECG, electrocardiogram; EDA, Electrodermal Activity; HR, Heart Rate; M, Mean; N, Numerosity of the sample; ND, Non‐Disclosed; PCE, Proportional Constant Error; PZG, Piezoelectrography; RSP, Respiration Rate; SCR, Skin Conductance Response; SD, Standard Deviation; VAS, Visual Analogue Scale; ↑, Increase; +, More Accurate; /, No Effect; <, Underestimation; >, Overestimation.

**TABLE 5:** Studies that used multimodal stimuli included in the emotion manipulation category.

Article	Sample *N* (Females)	Age M (SD)	Study design	Country	Arousal manipulation	Recording	Task	Durations	Arousal Index	Effects arousal	Time index	Effects time
Gros et al. (2015)	50 (27)	75 (ND)	Between	France	Odors	EDA	VAS	0.4–1.6 s	SC	↑	Δ post‐pre exposure	>
					Video					↑		/
Schreuder et al. (2014)	60 (45)	22.2 (3.4)	Between	Netherlands	Unrelaxing Position/Arousing Odor	ECG	Time Production	1.33–2.17 m	HR	↑	CE	<
					Unrelaxing Position/Arousing Odor	EDA	Time Production	1.33–2.17 m	SCR	↑		<
					Unrelaxing Position/Non‐Arousing Odor	ECG	Time Production	1.33–2.17 m	HR	↑		/
					Unrelaxing Position/Non‐Arousing Odor	EDA	Time Production	1.33–2.17 m	SCR	↑		/

Abbreviations: CE, constant error (subjective–objective); ECG, electrocardiogram; EDA, electrodermal activity; HR, heart rate; M, mean; N, numerosity of the sample; ND, non‐disclosed; SC, skin conductance; SCR, skin conductance response; SD, standard deviation; VAS, visual analogue scale; Δ, difference between raw estimates; ↑, increase; /, no effect; <, underestimation; >, overestimation.

#### 3.3.1 Emotional Images

The studies presented in this section show that the use of emotional images is associated with either greater temporal underestimation (Angrilli et al. 1997) or temporal overestimation (Ogden, Henderson, McGlone, and Richter 2019). An explanation for this discrepancy may be the different valence of the stimuli used. Angrilli et al. (1997) asked their participants to reproduce temporal intervals marked with images taken from the International Affective Picture System (IAPS; Lang et al. 1997) presented for 2–6 s, varying in valence and arousal (negative‐high, negative‐low, neutral, positive‐low, positive‐high, Figure 5A). Physiological arousal was assessed using ECG and EDA. The results showed that positive stimuli were associated with an increase in HR, while negative stimuli were associated with a significant decrease in HR. Furthermore, a significant increase in Skin Conductance Response (SCR) was observed for arousing stimuli, regardless of valence. Behaviourally, the results showed that high‐arousal positive stimuli were underestimated more than their negative counterparts. An opposite pattern was observed for low‐arousal stimuli, where negative stimuli were underestimated more than positive ones (Angrilli et al. 1997). Ogden, Henderson, McGlone, and Richter (2019) also used images from the IAPS that varied in their valence (neutral, negative, positive) and arousal (high vs. low), but with a verbal estimation task (0.2–0.8 s—Figure 5A). The authors found that emotional stimuli were perceived as lasting longer than neutral ones. While PNS activity, measured by HF‐HRV, was unaffected by valence or arousal, SNS activity, measured by PEP, increased with negative stimuli. For high‐arousal negative pictures, the increase in SNS activity was linked to longer perceived durations, but no such correlation was found for PNS activity. Positive stimuli showed no link between arousal and perceived duration. The role of PNS was further analyzed in another study by Ogden, Henderson, Slade, et al. (2019). In this study, the authors showed that increased PNS—manipulation obtained by instructing the participants to breathe at a controlled frequency of 0.1 Hz—was associated with systematic underestimation of time in a verbal estimation task (0.2–0.8 s—Figure 5A), regardless of the valence (negative vs. neutral) of the IAPS images used to mark probe stimuli.

**FIGURE 5:**
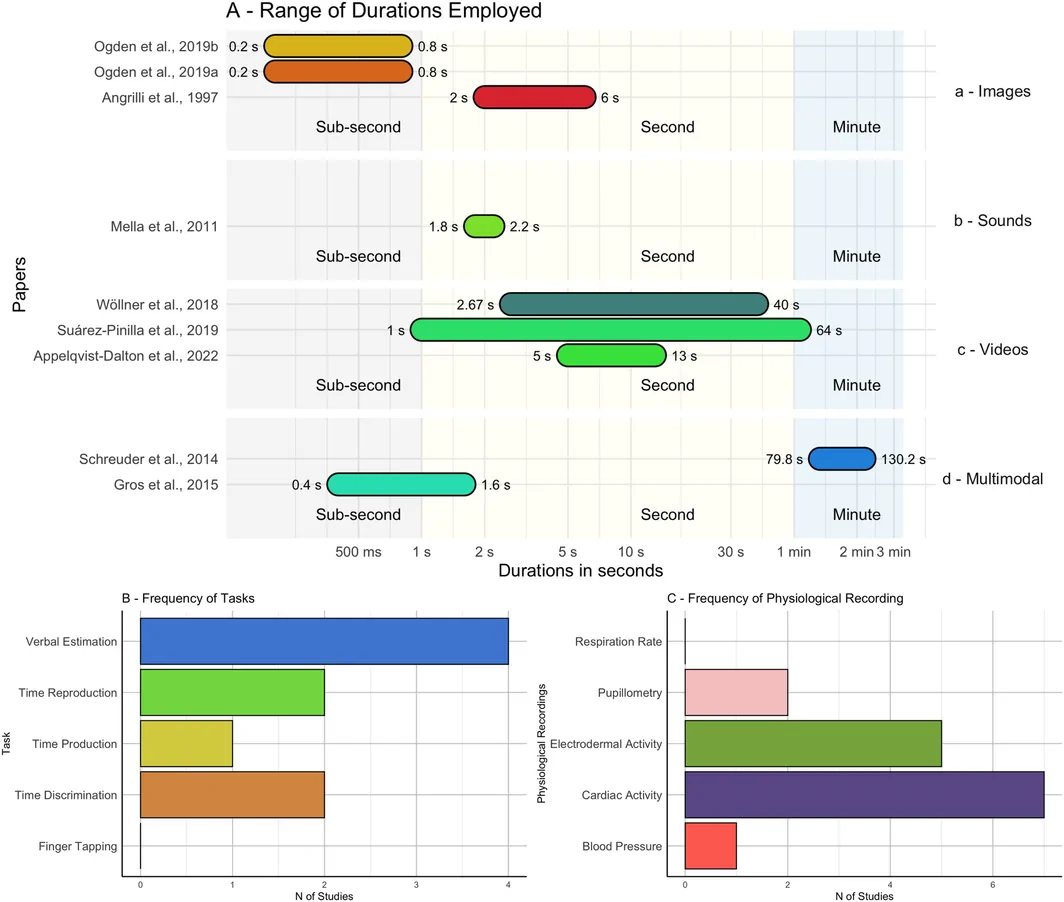
Duration ranges, frequency of tasks, and physiological measures in the analyzed studies. (A) Durations range from subsecond to minutes. (B) Number of studies using different timing tasks. (C) Frequency of physiological measures assessed.

Taken together, the results from these studies suggest that temporal distortions are shaped by both arousal and valence. More specifically, the activation of the SNS, particularly in combination with negative valence, appears to accelerate the internal pacemaker, leading to temporal overestimation during time estimation tasks (Ogden, Henderson, McGlone, and Richter 2019). However, in time reproduction tasks, SNS activation may instead lead to temporal underestimation, depending on the self‐reported arousal level used to select the stimuli (Angrilli et al. 1997). In contrast, activation of the PSN has been associated with shorter perceived durations (Ogden, Henderson, Slade, et al. 2019).

#### 3.3.2 Emotional Sounds

Only one of the selected studies has explored how auditory stimuli can evoke such physiological responses and influence time perception. Mella et al. (2011) asked their participants to complete a time discrimination task (1.8–2.2 s—Figure 5A) with auditory stimuli from the International Affective Digitized Sounds (IADS—Stevenson and James 2008) divided into neutral, high‐arousing unpleasant, and low‐arousing unpleasant sounds. The researchers also manipulated task demands by instructing participants to focus on different features of the stimuli: their emotional intensity, duration, or both. The findings revealed that when participants focused their attention on the emotion, they perceived the duration of high‐arousal sounds as significantly longer than that of neutral or low‐arousal sounds. Additionally, SCR, recorded as a physiological marker of arousal, was higher for high‐arousal sounds compared to other types of sounds. This study, in line with the ones that employed emotional images (Angrilli et al. 1997; Ogden, Henderson, McGlone, and Richter 2019; Ogden, Henderson, Slade, et al. 2019), supports the assumption that a combination of negative valence and SNS activation is associated with the tendency to judge durations as longer. Of note, the literature on temporal processing, emotional auditory stimuli, and physiological measures of arousal largely neglected positively valenced stimuli.

#### 3.3.3 Videos

Emotional videos represent another type of manipulation within this category. In this case, researchers have varied different aspects of the stimuli, such as complexity (Suárez‐Pinilla et al. 2019), speed (Wöllner et al. 2018), or sensory modality (Appelqvist‐Dalton et al. 2022), often aiming at triggering a response that goes beyond mere stimulus valence.

In their study, Appelqvist‐Dalton et al. (2022) asked participants to estimate the duration of video clips (5.2–12.13 s—Figure 5A) presented in three sensory modalities: Audio Only, Video Only, and Audio‐Video. Arousal was measured using self‐reports and pupil dilation. Results showed that all clips were perceived as shorter than their actual duration. However, higher arousal, indexed both by post‐stimulus pupil dilation and a composite measure combining subjective ratings and physiological responses during clip exposure, was associated with more accurate time estimates. Still, it was not possible to disentangle the unique contributions of physiological and subjective arousal during the test phase. Wöllner et al. (2018) studied how slow‐motion and real‐time speeds, along with music, affect time perception and psychophysiological responses. Participants watched clips of films, sports, or ballet and estimated their durations (2.67–40 s—Figure 4A) while their ECG, EDA, respiration, and ocular activity were recorded. The results showed that the durations of slow‐motion videos were underestimated. This condition was also associated with a lower respiration rate and pupil dilation compared to videos presented at normal speed. However, across conditions, the presence of music was generally linked to higher arousal levels. This hints at the importance of the influence of motion and speed on subjective time perception. Suárez‐Pinilla et al. (2019) asked participants to view dynamic videos (1–64 s, Figure 4A) and estimate their duration. The videos, depicting scenes like city walks, countryside exploration, and stationary environments, varied in the amount of perceptual change. Physiological measures (ECG, EDA, and oculometry) were recorded during the timing task. The results did not show any significant relationship between physiological arousal and temporal performance. Rather, temporal overestimation was significantly correlated with the amount of perceptual change.

Taken together, the results presented in this subsection suggest that the temporal distortion caused by video is based on the stimulus characteristics (i.e., sensory modality presented) in combination with other factors (i.e., speed of the video). Indeed, it seems that the elements that compose these combinations tend to conflict with each other, with the most “powerful” dominating the perceived duration, as in the case of perceptual complexity (Suárez‐Pinilla et al. 2019).

#### 3.3.4 Multimodal Stimuli

Studies included in this category used several types of stimuli, such as emotional images, videos, or images, combined with other arousing stimuli, like stressful experiences or uncomfortable positions. Schreuder et al. (2014) studied the effects of different olfactory stimuli on a production task (1.33–2.17 m—Figure 5A) while participants either remained upright (unrelaxed) or seated (relaxed). Exposure to more arousing odors led to shorter time productions compared to the no odor condition. However, this effect was not linked to physiological arousal, as neither HR nor EDA showed significant changes. Although body posture influenced arousal levels (with upright posture linked to higher arousal), it did not affect perceived time duration. Following this study, Gros et al. (2015) explored whether and how emotional stimuli from video and olfactory modalities affect time perception using an auditory temporal bisection task (0.4, 1.6 s—Figure 5A). Participants estimated the duration of neutral sounds after exposure to emotionally charged odors (pleasant, unpleasant, neutral) or video clips (positive, negative, neutral). Olfactory stimuli, particularly emotionally charged odors, increased EDA and led to overestimations of time. Video stimuli had a less pronounced effect, causing temporal underestimation for shorter intervals and overestimation for longer ones. Finally, we can also include in this category the already mentioned study by Van Hedger et al. (2017) in which the authors found that inducing stress reduced PNS activity and led to greater temporal distortions, with negative emotional stimuli perceived as lasting longer.

Taken together, the results presented in this subcategory corroborate the hypothesis that stimulus characteristics (i.e., in this case, sensory modality—Gros et al. 2015; Schreuder et al. 2014) modulate temporal distortions. Indeed, emotionally driven manipulations of arousal can lead to different temporal outcomes based on an experiment's characteristics(i.e., durations, sensory modality of the manipulation, and temporal tasks).

### 3.4 Studies With Arousal Manipulation Made With Physical Activity

Another arousal manipulation is physical activity, which can be used before, during, or after temporal tasks (Table 6). Schaefer and Gilliland (1938) asked participants to verbally estimate durations ranging from 4 to 27 s (Figure 6A), both before and after engaging in physical exercise (running up and down steps). During the experiment, they collected HR, respiration, and blood pressure. The results showed no significant effect of physical activity on temporal estimates. Schönpflug (1966) presented participants with a 15 s interval and then asked them to rate its duration on a 10‐point Likert‐type scale, ranging from “very short” to “very long,” while they were seated in a comfortable chair or engaged in physical activity on an ergometer. The temporal interval to be judged was presented in one of two ways: either by counting a series of digits and letters or by listening to a buzzer. As expected, physical activity heightened participants' arousal levels, as shown by increased skin resistance (SR). The effect on the temporal task depended on the presentation modality of the stimulus, with temporal overestimation in the listening condition and temporal underestimation in the counting condition. Furthermore, SR enhanced the effect by increasing the overestimation or underestimation in the physical activity condition compared to the control condition. Curton and Lordahl (1974) manipulated participants' arousal using exercise or by threatening them with electric shock while they were asked to estimate the duration of a secondary task (5–19 s—Figure 6A). The results showed that HR decreased in the exercise group, while being exposed to the threat of shock caused HR to either stabilize or increase. Additionally, exercise‐induced arousal negatively correlated with time estimation accuracy, while shock threat‐induced arousal showed no significant correlation, indicating that the nature of the stimulus (physical vs. stressful) can be a modulator of the relationship in the exam. Vercruyssen et al. (1989) asked participants to produce 10‐s intervals before, during, and after engaging in acute physical exercise. The participants cycled on a cycle ergometer at moderate and high intensities, with intensity assessed through oxygen consumption. Their findings revealed that time production was less consistent during the exercise phase compared to the pre‐ and post‐exercise phases, suggesting that acute physical activity—and the resulting increase in physiological arousal (HR)—can disrupt accurate time estimation. Building on this, Lambourne (2012) examined how cycling or resting before and after a temporal discrimination task (0.142–1.270 s—Figure 6A) influenced time perception. The results showed that after cycling, participants tended to overestimate these durations, with physiological arousal measured by HR being higher after physical exercise. Schwarz et al. (2013) manipulated physiological and self‐reported measures of arousal in three conditions: muscular exercise, breath‐holding, or a control condition with no additional task. During these, participants estimated the duration of symbolic figures shown on a screen for 8 s. Muscular exercise significantly increased HR compared to the other conditions, while both muscular exercise and breath‐holding conditions led to higher levels of subjective arousal than the control condition. Notably, participants in both the muscular exercise and breath‐holding conditions judged the stimulus durations as longer than those in the control condition. This suggests that subjective arousal, rather than physiological arousal, is what drives temporal overestimation. These results were confirmed in a second experiment by the same group, where they used a time production task (10 s—Figure 6A) instead of time estimation, strengthening the conclusion that subjective arousal plays a central role in shaping time perception. Dormal et al. (2018) asked participants to perform a temporal categorization task (0.5–0.9 s—Figure 6A) before and after arousal manipulations. Participants were divided into three groups: one cycled on a cycle ergometer, another engaged in guided muscular relaxation, and a third completed a low‐arousal activity such as solving crosswords. As a result, cycling increased HR and relaxation reduced it compared to the crossword condition. However, after the manipulations, participants overestimated durations in all conditions, suggesting that the physiological changes alone did not account for the differences in time perception. The authors proposed that this general overestimation might be due to interference from the intervening tasks and a delay between the pre‐ and post‐manipulation phases. A subsequent experiment confirmed this explanation, indicating that physical arousal by itself does not have a direct effect on temporal processing.

**TABLE 6:** Studies included in the physical activity category.

Article	Sample *N* (Females)	Age M (SD)	Study design	Country	Arousal manipulation	Recording	Task	Durations	Arousal index	Effects arousal	Time index	Effects time
Curton and Lordahl (1974)	60 (22)	ND	Between	ND	Anxiety—TR	ECG	Verbal Estimation	5–19 s	HR	↑	RE	/
					Anxiety—TI					↑		/
					Physical—TR					↑		—
					Physical—TI					↑		—
Dormal et al. (2018)	18 (9)	28 (3.5)	Within	Belgium	Cycling	ECG	Temporal Categorization	0.5–0‐9 s	HR	↑	RL	/
					Relaxing					↓		/
					Crosswords					/		/
Lambourne (2012)	18 (8)	21.06 (0.84)	Within	ND	Cycling	ECG	Temporal Generalization	0.142–1.270 s	HR	↑	P(YES)	/
Schaefer and Gilliland (1938)	4 (0)	ND	Within	ND	Running	ECG	Verbal Estimation	4–27 s	HR	/	Percentage of Over‐ and Under‐ estimation	/
					Running			4–27 s	Heart Work	/		/
					Running	PNG		4–27 s	RSP	/		/
					Running			4–27 s	Resp. Work	/		/
					Running	BPR		4–27 s	BPR	/		/
Schönpflug (1966)	60 (ND)	ND	Between	ND	Cycling—Counting	EDA	VAS	15 s	SR	↑	RE	>
					Cycling—Listening			15 s		↑		>
					Relaxing—Counting			15 s		↑		/
					Relaxing—Listening			15 s		↑		/
Schwarz et al. (2013)	30 (20)	25.5 (6.5)	Within	Germany	Muscular Exercise	ECG	Verbal Estimation	8 s	HR	↑	RE	/
					Breath Hold					↑		/
	30 (23)	21.7 (2.7)	Within	Germany	Muscular Exercise	ECG	Time Production	8 s	HR	↑	RE	/
					Breath Hold					↑		/
Vercruyssen et al. (1989)	11 (0)	25.0 (2.6)	Within	ND	Cycling	ECG	Time Production	10s	HR	↑	RE	—

Abbreviations: BPR, blood pressure; ECG, electrocardiogram; EDA, electrodermal activity; HR, heart rate; M, mean; N, numerosity of the sample; ND, non‐disclosed; P(YES), proportion of yes responses; PNG, pneumograph; RE, raw estimate; Resp, respiration; RL, response latencies; RSP, respiration rate; SD, standard deviation; SR, skin resistance; TI, time irrelevant; TR, time relevant; VAS, visual analogue scale; ↑, Increase; ↓, Decrease; −, Less Accurate; /, No Effect; >, Overestimation.

**FIGURE 6:**
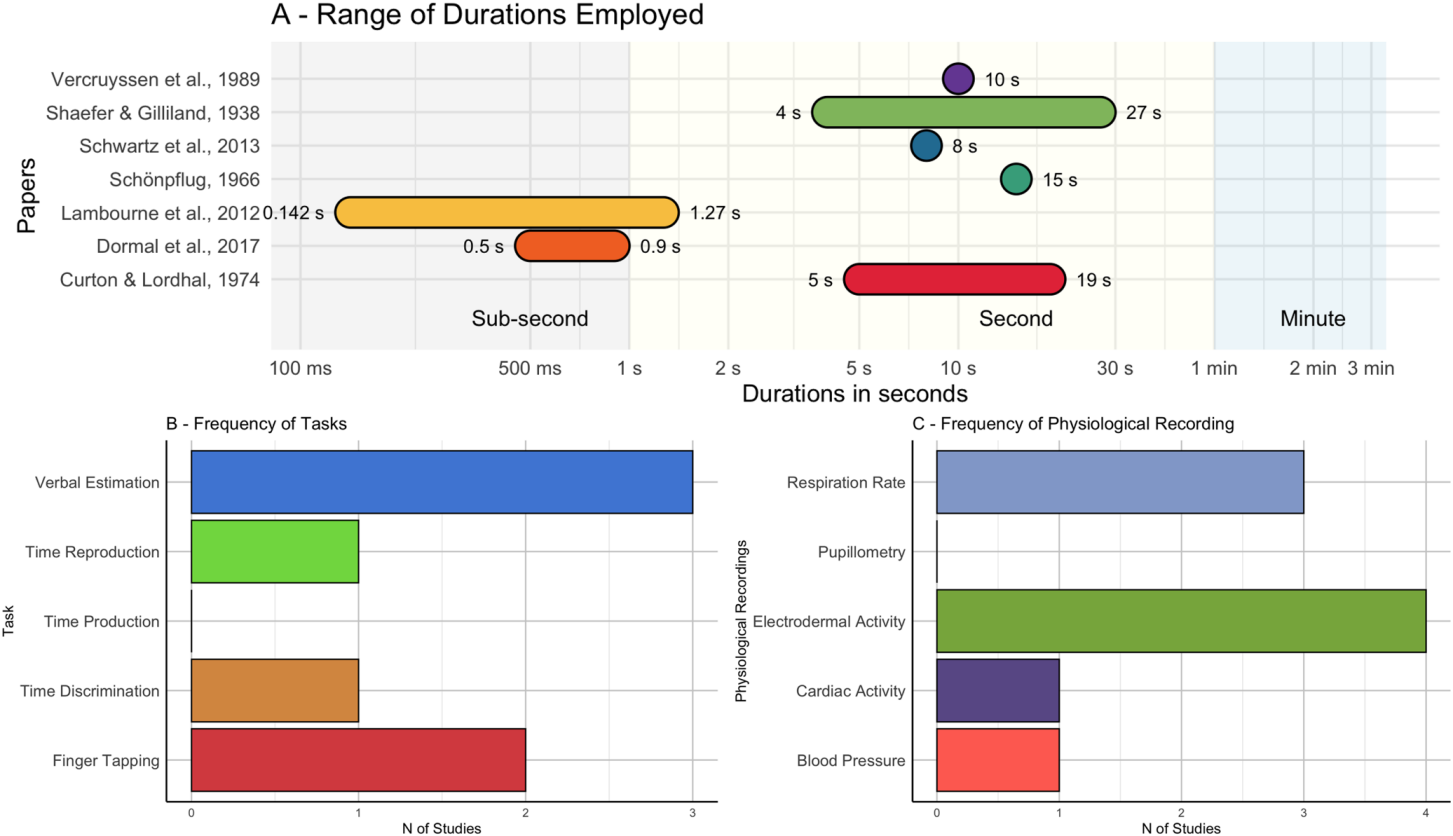
Duration ranges, frequency of tasks, and physiological measures in the analyzed studies. (A) Durations range from subsecond to minutes. (B) Number of studies using different timing tasks. (C) Frequency of physiological measures assessed.

In summary, while physical activity and physiological arousal can influence time perception, the direction and magnitude of these effects are shaped by a complex interplay of factors. These include the type of timing task used (Figure 6B), the timing and nature of concurrent cognitive demands, the delay between the arousal manipulation and the timing task, and the specific physiological measures recorded (Figure 6C). Additionally, subjective experiences of arousal and the level of cognitive engagement also contribute to how duration is perceived.

## 4 Conclusions

The human ability to perceive time, though central to daily life, does not always align with the objective passage of time. This temporal distortion is often attributed to changes in arousal. While extensive research has highlighted a connection between arousal and time perception, the nature of this relationship remains poorly understood. The primary aim of this work was to investigate how physiological arousal contributes to temporal distortions, operationalizing arousal in terms of autonomic nervous system (ANS) activation across a broad range of temporal tasks, arousal manipulations, and physiological measures. Overall, the reviewed evidence indicates that physiological arousal is related to temporal processing, but this relationship is not uniform and cannot be adequately described as a simple association between the magnitude of physiological activation and the direction and/or magnitude of temporal distortion. A relatively consistent association was observed between baseline cardiac autonomic activity and temporal accuracy, particularly for parasympathetic activity indexed by heart rate variability (HRV). In contrast, when arousal was experimentally manipulated, changes in physiological activity were not consistently associated with either overestimation or underestimation of time. Instead, the temporal consequences of arousal appeared to depend on several characteristics of the eliciting experience, including its emotional and perceptual properties, relevance to the temporal task, sensory modality, cognitive demands, and subjective experience. Thus, the findings suggest that physiological arousal may be involved in temporal processing without being sufficient, on its own, to account for temporal distortions.

To fulfill our aims, we focused on studies that measured physiological indicators of arousal, such as ECG, EDA, eye‐tracking, and respiration rate. Among these, ECG stands out as the most frequently used, appearing in 81% of the total studies. Within ECG recordings, HR was the predominant index computed as a measure of arousal. A second widely used index was HRV, typically expressed as HF‐HRV, LF‐HRV in the frequency domain, or RMSSD in the time domain.

When considering the association between these indices and performance on temporal tasks, no consistent conclusions can be drawn. This inconsistency may be due to several factors related to the experimental manipulations (e.g., type and duration of the manipulation), the task characteristics, and, most critically, the duration of the intervals under investigation. Indeed, many studies focusing on cardiac activity employed very short temporal intervals (ranging from milliseconds to a few seconds), and thus typically recorded physiological data during resting states (e.g., Cellini et al. 2015, see tab. 1) or the manipulation phases (e.g., Osato et al. 1995, see tab 2), but rarely during task execution itself. Although cardiac activity and variability indices dominate the field in terms of usage, we emphasize that such measures are not well‐suited for studying very brief intervals, since the recording durations useful to extract that information are much longer (Shaffer and Ginsberg 2017). This leaves a major open question, as we currently lack insight into what occurs while participants are actively processing the temporal interval, at least from an autonomic point of view.

A second physiological index used to assess arousal in relation to time perception is EDA, which was employed in 41% of the total of the studies. Although EDA has been associated with both overestimations (e.g., Mella et al. 2011) and underestimations of time (e.g., Wöllner et al. 2018), it appears to function as a booster of temporal distortion. In other words, higher levels of EDA are often linked to a stronger effect of the experimental manipulation. Also in this case, duration intervals are a concern for a proper understanding of the relationship between EDA and time processing. Indeed, due to the intrinsic nature of EDA, which responses unfold in seconds, only the studies that employed longer durations were able to assess this measure during the task execution (e.g., Meissner and Wittmann 2011), while other studies used EDA to test the effectiveness of the manipulation employed (e.g., Cellini et al. 2023).

Respiration activity was another measure recorded in some of the studies presented (13% of the total number of studies). However, no clear relationship with temporal processing was observed. Indeed, besides a significant correlation with the speed of video stimuli (Wöllner et al. 2018), no other significant correlations were reported in the studies we examined in this systematic review. This result may be explained by the fact that the video content in the study by Wöllner et al. (2018)—ballet and sports—naturally promotes synchronization between internal rhythms and external sensory input, thereby producing the observed effect.

Oculometry was employed in fewer than 10% of the total number of studies. Two studies (Appelqvist‐Dalton et al. 2022; Wöllner et al. 2018) used pupillometry, and one study investigated eye movements (Suárez‐Pinilla et al. 2019). Due to methodological heterogeneity in how pupillometric indices were extracted—for example, Appelqvist‐Dalton et al. (2022) used a composite measure of arousal combining pupillometry parameters with self‐reported perception of arousal—as well as differences in experimental manipulations, it was not possible to draw definitive conclusions from the pupillometry studies. Nevertheless, pupillometry represents an excellent tool to overcome the issues related to studying very brief intervals, given its sensitivity to rapid fluctuations in arousal and cognitive load (Skaramagkas et al. 2023).

Interestingly, the relationship between physiological arousal and temporal processing seems to depend on how this association is assessed. For instance, the studies that did not actively manipulate arousal but rather measured it at baseline showed a stronger relation with arousal, with HRV being associated with more accurate performances. This suggests that HR may serve as a timekeeper, as higher HRV is associated with more accurate temporal performances. However, this speculation is no longer supported by research data when arousal is actively manipulated in experimental settings. Indeed, across studies involving pain, stress, emotional stimuli, and physical activity, physiological changes were not consistently accompanied by a predictable temporal distortion. This observation is important because it challenges a straightforward interpretation in which an increase in physiological arousal would accelerate or slow an internal timing mechanism in a uniform manner. But rather, it suggests the possible implication of other elements. For instance, the findings from studies involving emotional stimuli provide further evidence that the effects of arousal cannot be separated from the characteristics of the stimulus that elicits it. Angrilli et al. (1997) showed that positive and negative emotional stimuli produced different cardiac responses, while electrodermal responses were more closely related to arousal irrespective of valence. Importantly, the temporal effects differed as a function of both valence and arousal. Similarly, Ogden, Henderson, McGlone, and Richter (2019) found that emotional stimuli were perceived as longer than neutral stimuli, but that the relationship between physiological measures and temporal judgments depended on the emotional characteristics of the stimuli. The same conclusion is supported by studies using auditory stimuli. Mella et al. (2011) found that highly arousing sounds were judged as longer, particularly when attention was directed toward their emotional characteristics, and that these stimuli elicited greater skin conductance responses. Studies using dynamic videos similarly showed that temporal judgments depended on multiple interacting stimulus characteristics. Wöllner et al. (2018) found that slow‐motion videos were underestimated relative to real‐time videos and that changes in physiological and ocular measures accompanied the different viewing conditions. And Appelqvist‐Dalton et al. (2022) found that higher arousal was associated with more accurate estimates, but the independent contributions of subjective and physiological arousal could not be clearly separated. Multimodal studies reinforce this complexity. Schreuder et al. (2014) found that arousing odors affected time production without a corresponding relationship with physiological arousal, while posture increased physiological arousal without producing a comparable temporal effect. Gros et al. (2015) reported that emotional odors increased electrodermal activity and temporal overestimation, whereas video stimuli produced different effects depending on the duration being judged. Thus, even when an experimental manipulation reliably alters physiological arousal, the temporal consequences appear to depend on what participants are experiencing, how the experience is processed, and what they are required to do with respect to time. This observation raises the question of what determines whether a given physiological response is accompanied by temporal distortion, increased accuracy, or no temporal effect. One possibility is that the physiological response is embedded within a broader process involving the evaluation of the eliciting event. In this context, appraisal theories provide a potentially useful theoretical framework. Appraisal refers to the cognitive evaluation of a situation in relation to its relevance to one's goals, well‐being, and coping resources and plays a central role in shaping emotional and physiological responses (Lazarus 1991; Scherer 2001). Applied to time perception, appraisal may help explain why similar physiological responses can have different consequences for temporal judgments. An event that is perceived as threatening, relevant, unexpected, or personally significant may recruit attention and cognitive resources differently from an event that produces a comparable physiological response but is perceived as less relevant. Several findings in the present review are compatible with this interpretation. The contrast between electrical and thermal stimulation reported by Piovesan et al. (2019) is particularly informative because comparable subjective pain did not result in comparable temporal effects. Furthermore Schwarz et al. (2013) found that subjective arousal was more closely related to temporal overestimation than physiological arousal, while Dormal et al. (2018) showed that physiological changes could occur without a corresponding specific temporal effect. Importantly, this interpretation should not be taken as evidence that appraisal has been demonstrated as the mechanism underlying the reviewed effects. The studies included in the present review were not designed to systematically test appraisal, and most did not directly measure or manipulate appraisal processes. Rather, appraisal emerges as a candidate explanatory framework from the qualitative synthesis of the available evidence.

Another important reflection should be made on the diversity of time perception tasks used—including verbal estimation, reproduction, production, comparison, and finger tapping—each of which engages different cognitive and neural mechanisms and offers differential sensitivity to internal clock alterations. Crucially, the choice of duration ranges (from milliseconds to several minutes) also plays an important role, as different mechanisms may underlie the processing of sub‐second vs. supra‐second intervals (Lewis and Miall 2003).

Together, these inconsistencies make it challenging to draw a unified conclusion about the nature of the effects of arousal on time perception. However, heterogeneity is not confined to the methodological choices regarding temporal estimation tasks; rather, it represents a pervasive feature of the entire field investigating the relationship between arousal and time perception. One of the most prominent sources of variability lies in the experimental manipulations used to induce arousal. These manipulations differ not only across broad categories—such as emotional or physiological interventions—but also within the same category. Taking emotional manipulation as an example, we find significant differences in the sensory modality employed (e.g., visual, auditory, and multimodal), in the specific types of stimuli used (e.g., images, film clips, and sounds), and in the affective characteristics of these stimuli, such as their valence or intensity. Moreover, certain manipulations transcend emotional content altogether by introducing variations in perceptual complexity (Suárez‐Pinilla et al. 2019) or presentation speed (Wöllner et al. 2018), all of which can independently influence time perception. This level of intra‐category heterogeneity adds an additional layer of complexity to an already fragmented literature. Such inconsistency in arousal induction paradigms, when combined with the aforementioned diversity in timing tasks and duration ranges, has posed a significant obstacle to synthesizing findings across studies. In particular, it made any attempt to conduct a meta‐analysis unfeasible. Indeed, it was extremely difficult to identify studies with comparable methodological frameworks, especially when considering work conducted outside of the same research group, where subtle but critical differences in procedures, task demands, or stimulus selection often arise. This lack of standardization not only limits the generalizability of findings but also hampers the development of a unified theoretical model capable of capturing the nuanced interplay between arousal and temporal cognition.

The findings of this review suggest several priorities for future research. First, physiological arousal, subjective arousal, and appraisal should be measured separately and simultaneously. The distinction is important because subjective arousal does not necessarily correspond directly to temporal distortion, as illustrated in the present review. Designs combining autonomic measures with explicit assessments of subjective experience could help determine whether temporal distortions are more closely associated with physiological activation, subjective arousal, appraisal, or their interaction. Second, future studies should manipulate characteristics of the eliciting event systematically. Valence, perceived threat, relevance, novelty, perceptual complexity, and cognitive demands could be varied independently while physiological and subjective responses are recorded. Such designs would provide a more direct test of whether appraisal‐related processes explain variability in the temporal consequences of physiological arousal. Third, multimodal physiological assessment should become more common. Combining cardiac, electrodermal, respiratory, and ocular measures would allow researchers to determine whether and which different physiological components contribute differently to temporal processing. Fourth, future work should pay greater attention to the temporal task and duration being studied. Direct comparisons between different timing paradigms and temporal scales within the same participants would help establish whether the effects of arousal depend on the cognitive requirements of the temporal judgment. Finally, greater ecological validity may be particularly important. Real‐world arousing experiences are rarely isolated changes in physiological activation. They are dynamic, multisensory, emotionally meaningful, and embedded within ongoing cognitive and social contexts. Understanding how such experiences alter temporal perception may therefore require experimental paradigms that reproduce some of this contextual complexity while retaining sufficient experimental control.

To conclude, the present systematic review indicates that physiological arousal is related to temporal processing, but the available evidence does not support a simple or invariant relationship between physiological activation and temporal distortion. Baseline cardiac autonomic activity, particularly parasympathetic activity indexed by HRV, shows a relatively consistent association with temporal accuracy. However, when arousal is experimentally manipulated, changes in physiological activity are not consistently associated with the direction or magnitude of temporal distortions. Instead, temporal effects vary according to the characteristics of the eliciting event, the temporal task, the duration being judged, and probably the individual's subjective experience. The heterogeneity of these findings therefore represents more than a limitation of the existing literature. It suggests that physiological arousal may be one component of a broader process rather than a sufficient mechanism for explaining changes in subjective time. Within this framework, appraisal emerges as a theoretically grounded candidate for understanding how the characteristics and perceived significance of an arousing event may shape the relationship between physiological activation and temporal processing. This interpretation is not directly tested by the present review and should therefore be regarded as a hypothesis generated by the qualitative synthesis. Future studies specifically examining the interplay between appraisal, subjective arousal, physiological activation, and temporal processing will be necessary to determine whether this framework can account for the variability observed across the literature.

## Author Contributions

**Luigi Micillo:** conceptualization, methodology, software, formal analysis, investigation, data curation, visualization, writing – original draft, writing – review and editing. **Nicola Cellini:** conceptualization, methodology, validation, resources, supervision, writing – review and editing. **Giovanna Mioni:** conceptualization, methodology, validation, resources, supervision, writing – review and editing.

## Funding

The authors have nothing to report.

## Conflicts of Interest

The authors declare no conflicts of interest.

## Data Availability

Data sharing not applicable to this article as no datasets were generated or analysed during the current study.
